# Poly[1,4-bis­(4-pyridylmeth­yl)piperazine­diium [[tetra­aqua­cobaltate(II)]-μ-pyromellitato-κ^2^ *O* ^1^:*O* ^4^] dihydrate]

**DOI:** 10.1107/S1600536809051101

**Published:** 2009-11-28

**Authors:** Laura K. Sposato, Robert L. LaDuca

**Affiliations:** a Department of Chemistry and Physics, King’s College, Wilkes-Barre, PA 18711, USA; b Lyman Briggs College, Department of Chemistry, Michigan State University, East Lansing, MI 48825, USA

## Abstract

In the title compound, {(C_16_H_22_N_4_)[Co(C_10_H_2_O_8_)(H_2_O)_4_]·2H_2_O}_*n*_, the octa­hedrally coordinated Co^II^ atom is situated on an inversion center and possesses four aqua ligands. The Co atoms are linked into an anionic coordination polymer chain by bis-monodentate pyromellitate ligands. The chain motifs are connected into a supra­molecular layer by hydrogen bonding mediated by uncoordinated water mol­ecules. Charge balance is provided by doubly protonated bis­(4-pyridylmeth­yl)piperazine units, which are anchored to the coordination polymer chain motifs by N—H⋯O hydrogen bonding.

## Related literature

For some divalent cobalt pyromellitate coordination polymers containing dipyridyl ligands, see: Majumder *et al.* (2006 ▶). For the preparation of bis­(4-pyridylmeth­yl)piperazine, see: Pocic *et al.* (2005 ▶).
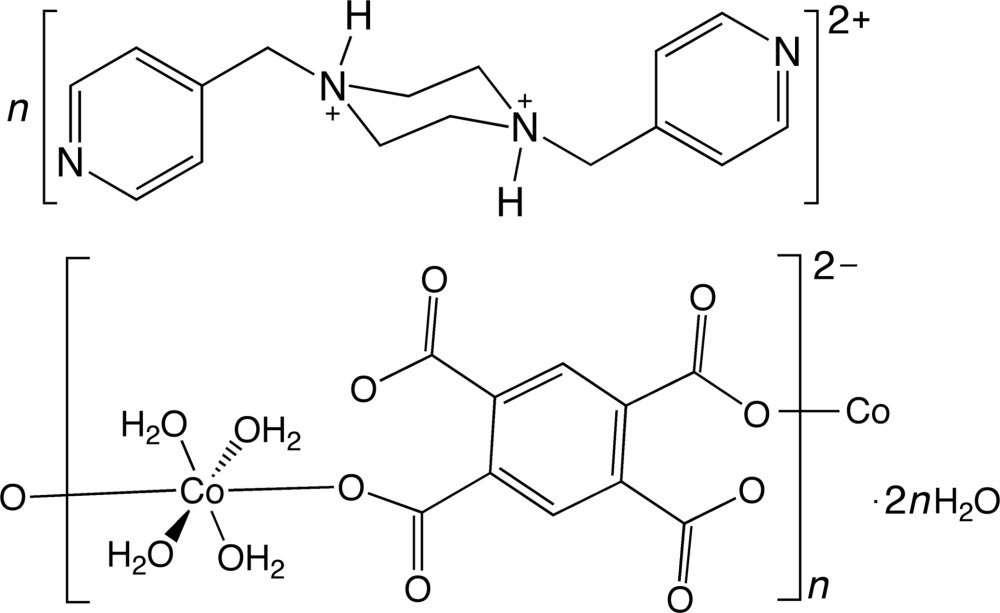


## Experimental

### 

#### Crystal data

(C_16_H_22_N_4_)[Co(C_10_H_2_O_8_)(H_2_O)_4_]·2H_2_O
*M* *_r_* = 687.52
Triclinic, 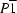

*a* = 7.278 (2) Å
*b* = 9.752 (3) Å
*c* = 11.257 (3) Å
α = 66.733 (3)°
β = 75.168 (3)°
γ = 83.359 (3)°
*V* = 709.5 (3) Å^3^
*Z* = 1
Mo *K*α radiation
μ = 0.69 mm^−1^
*T* = 173 K
0.24 × 0.14 × 0.10 mm

#### Data collection

Bruker APEXII CCD diffractometer
Absorption correction: multi-scan (*SADABS*; Sheldrick, 1996 ▶) *T*_min_ = 0.853, *T*_max_ = 0.933
11370 measured reflections
2908 independent reflections
2511 reflections with *I* > 2σ(*I*)
*R*_int_ = 0.058

#### Refinement

*R*[*F* ^2^ > 2σ(*F* ^2^)] = 0.050
*wR*(*F* ^2^) = 0.153
*S* = 1.05
2908 reflections
226 parameters
10 restraints
H atoms treated by a mixture of independent and constrained refinement
Δρ_max_ = 1.23 e Å^−3^
Δρ_min_ = −0.91 e Å^−3^

### 

Data collection: *APEX2* (Bruker, 2007 ▶); cell refinement: *SAINT* (Bruker, 2007 ▶); data reduction: *SAINT*; program(s) used to solve structure: *SHELXS97* (Sheldrick, 2008 ▶); program(s) used to refine structure: *SHELXL97* (Sheldrick, 2008 ▶); molecular graphics: *CrystalMaker* (Palmer, 2007 ▶); software used to prepare material for publication: *SHELXL97*.

## Supplementary Material

Crystal structure: contains datablocks I, global. DOI: 10.1107/S1600536809051101/hy2258sup1.cif

Structure factors: contains datablocks I. DOI: 10.1107/S1600536809051101/hy2258Isup2.hkl

Additional supplementary materials:  crystallographic information; 3D view; checkCIF report

## Figures and Tables

**Table 1:** Hydrogen-bond geometry (Å, °)

*D*—H⋯*A*	*D*—H	H⋯*A*	*D*⋯*A*	*D*—H⋯*A*
O1*W*—H1*WA*⋯O6^i^	0.88 (2)	2.38 (3)	2.997 (3)	128 (3)
O1*W*—H1*WB*⋯O1^i^	0.89 (2)	1.88 (2)	2.764 (3)	174 (3)
O5—H5*A*⋯N1	0.88 (2)	1.87 (2)	2.739 (3)	177 (3)
O5—H5*B*⋯O4	0.85 (2)	1.87 (2)	2.697 (3)	163 (3)
O6—H6*C*⋯O1*W*	0.86 (2)	1.92 (2)	2.753 (3)	165 (3)
O6—H6*D*⋯O2^ii^	0.86 (2)	1.81 (2)	2.624 (3)	158 (3)
N2—H2*N*⋯O3^iii^	0.91 (2)	1.73 (2)	2.630 (3)	171 (3)

Symmetry codes: (i) 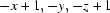; (ii) 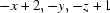; (iii) 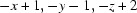.

**Fig. 1.**
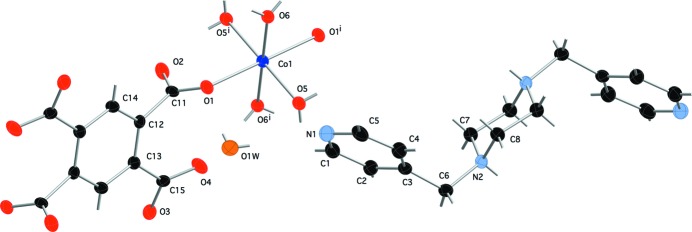
The coordination environment of the title compound, showing 50% probability ellipsoids. H atom positions are shown as grey sticks. [Color codes: dark blue Co; light blue N; red O; black C; orange O in uncoordinated water molecule. Symmetry code: (i) -x+2, -y, -z+1.]

**Fig. 2.**
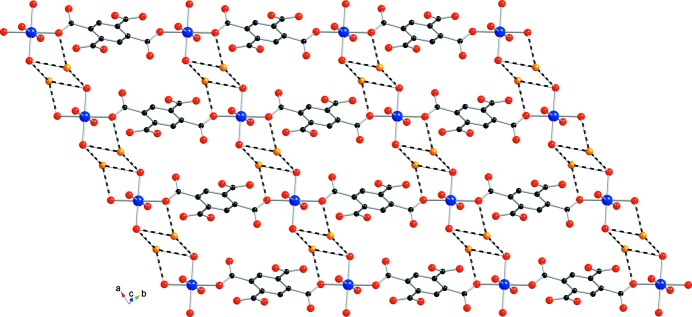
A view of the supramolecular layer in the title compound. Hydrogen bonding contacts are indicated as dashed bars.

**Fig. 3.**
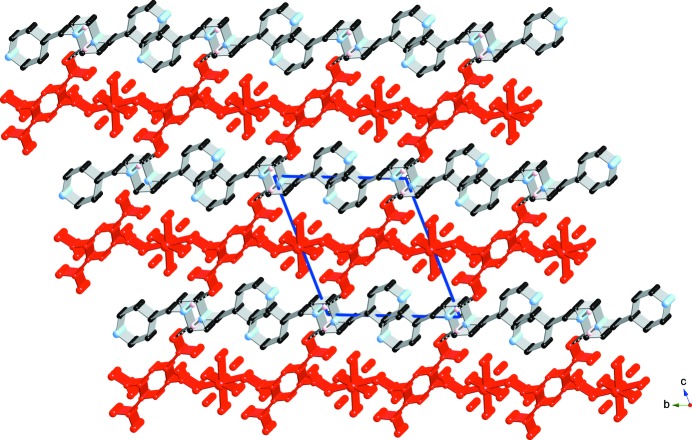
Stacking diagram for the title compound, viewed down the a axis. Hydrogen bonding contacts are indicated as dashed bars.

## supplementary crystallographic information

### Comment

The diverse possible binding modes of the pyromellitate ligand (1,2,4,5-benzenetetracarboxylate) has allowed formation of a wide variety of cobalt-containing coordination polymers, especially in the presence of dipyridyl neutral co-ligands (Majumder *et al.*, 2006). This chemistry was further developed by the synthesis of the title compound, which incorporates the long-spanning hydrogen-bonding capable dipyridyl ligand bis(4-pyridylmethyl)piperazine (bpmp).

The asymmetric unit of the title compound consists of a divalent Co^II^ atom on a crystallographic inversion center, one-half of a pyromellitate tetraanion situated across another crystallographic inversion center, one-half of a (H_2_bpmp)^2+^ dication (protonated at each of the two piperazinyl N atoms) sited across another crystallographic inversion center, and one water molecule of crystallization. The local coordination and surrounding supramolecular environment is illustrated in Fig. 1.

Adjacent Co^II^ ions are linked into [Co(H_2_O)_4_(pyromellitate)]_n_^2n-^ anionic one-dimensional coordination polymer motifs, *via* symmetrically related monodentate carboxylate termini of the pyromellitate ligands. These chain motifs are oriented parallel to the [1 1 0] direction; the Co···Co distance along the chain is 11.474 (3) Å. Two of the pyromellitate carboxylate groups do not ligate to Co^II^ ions. Neighboring chain motifs aggregate into supramolecular layers coincident with the *ab* planes (Fig. 2), established by hydrogen-bonding patterns between the co-crystallized water molecules, aqua ligands, and ligated pyromellitate carboxylate O atoms (Table 1). In turn, the supramolecular layers stack in an *AAA* pattern along the *c* axis, with charge-balancing (H_2_bpmp)^2+^ dications situated in the interlamellar regions (Fig. 3), thus forming the three-dimensional crystal structure of the title compound. The closest Co···Co contact distance between neighboring layers is 11.257 (3) Å, which defines the *c* lattice parameter.

### Experimental

All starting materials were obtained commercially, except for bpmp, which was prepared by a published procedure (Pocic *et al.*, 2005). A mixture of cobalt nitrate hexahydrate (108 mg, 0.37 mmol), pyromellitic acid (94 mg, 0.37 mmol), bpmp (99 mg, 0.37 mmol) and 10.0 g water (550 mmol) was placed into a 23 ml Teflon-lined Parr Acid Digestion bomb, which was then heated under autogenous pressure at 393 K for 48 h. After cooling to 293 K, orange blocks of the title compound were obtained along with a white powder.

### Refinement

All H atoms bound to C atoms were placed in calculated positions and refined in riding mode, with C—H = 0.95 and 0.99 Å, and with *U*_iso_(H) = 1.2*U*_eq_(C). The H atoms bound to the water molecule O atoms and to the piperazinyl N atoms were found in a difference Fourier map and refined with restraints of O—H = 0.89 (1) and N—H = 0.92 (1) Å and with *U*_iso_(H) = 1.2*U*_eq_(O,N). The maximum and minimum residual electron density peaks of 1.234 and -0.908 e Å^-3^ were located 0.98 and 0.68 Å from the Co1 and O1W atoms, respectively.

### Crystal data

**Table:**

(C_16_H_22_N_4_)[Co(C_10_H_2_O_8_)(H_2_O)_4_]·2H_2_O	*Z* = 1
*M**_r_* = 687.52	*F*(000) = 359
Triclinic, *P*1	*D*_x_ = 1.609 Mg m^−^^3^
Hall symbol: -P 1	Mo *K*α radiation, λ = 0.71073 Å
*a* = 7.278 (2) Å	Cell parameters from 11370 reflections
*b* = 9.752 (3) Å	θ = 2.0–26.5°
*c* = 11.257 (3) Å	µ = 0.69 mm^−^^1^
α = 66.733 (3)°	*T* = 173 K
β = 75.168 (3)°	Block, pink
γ = 83.359 (3)°	0.24 × 0.14 × 0.10 mm
*V* = 709.5 (3) Å^3^	

### Data collection

**Table:**

Bruker APEXII CCD diffractometer	2908 independent reflections
Radiation source: fine-focus sealed tube	2511 reflections with *I* > 2σ(*I*)
graphite	*R*_int_ = 0.058
φ and ω scans	θ_max_ = 26.5°, θ_min_ = 2.0°
Absorption correction: multi-scan (*SADABS*; Sheldrick, 1996)	*h* = −9→9
*T*_min_ = 0.853, *T*_max_ = 0.933	*k* = −12→12
11370 measured reflections	*l* = −14→14

### Refinement

**Table:**

Refinement on *F*^2^	Primary atom site location: structure-invariant direct methods
Least-squares matrix: full	Secondary atom site location: difference Fourier map
*R*[*F*^2^ > 2σ(*F*^2^)] = 0.050	Hydrogen site location: inferred from neighbouring sites
*wR*(*F*^2^) = 0.153	H atoms treated by a mixture of independent and constrained refinement
*S* = 1.05	*w* = 1/[σ^2^(*F*_o_^2^) + (0.1134*P*)^2^] where *P* = (*F*_o_^2^ + 2*F*_c_^2^)/3
2908 reflections	(Δ/σ)_max_ < 0.001
226 parameters	Δρ_max_ = 1.23 e Å^−^^3^
10 restraints	Δρ_min_ = −0.91 e Å^−^^3^

### Fractional atomic coordinates and isotropic or equivalent isotropic displacement parameters (Å^2^)

**Table:**

	*x*	*y*	*z*	*U*_iso_*/*U*_eq_	
Co1	1.0000	0.0000	0.5000	0.0155 (2)	
O1	0.8311 (2)	0.19744 (19)	0.45730 (18)	0.0192 (4)	
O1W	0.4690 (3)	−0.1576 (2)	0.6340 (2)	0.0255 (4)	
H1WA	0.437 (4)	−0.070 (3)	0.639 (3)	0.031*	
H1WB	0.375 (4)	−0.178 (4)	0.607 (3)	0.031*	
O2	0.9191 (3)	0.2866 (2)	0.58976 (19)	0.0235 (4)	
O3	0.3143 (3)	0.2159 (2)	0.82962 (18)	0.0236 (4)	
O4	0.5304 (3)	0.1042 (2)	0.7189 (2)	0.0332 (5)	
O5	0.8431 (3)	−0.0690 (2)	0.69373 (18)	0.0183 (4)	
H5A	0.829 (4)	−0.163 (2)	0.748 (3)	0.022*	
H5B	0.742 (3)	−0.024 (3)	0.718 (3)	0.022*	
O6	0.8063 (3)	−0.0969 (2)	0.44473 (18)	0.0193 (4)	
H6C	0.705 (3)	−0.132 (3)	0.504 (3)	0.023*	
H6D	0.876 (4)	−0.166 (3)	0.425 (3)	0.023*	
N1	0.7991 (3)	−0.3648 (3)	0.8552 (2)	0.0284 (6)	
N2	0.8089 (3)	−0.9425 (2)	1.0284 (2)	0.0170 (5)	
H2N	0.755 (4)	−1.033 (2)	1.080 (3)	0.020*	
C1	0.7611 (4)	−0.4408 (3)	0.9870 (3)	0.0258 (6)	
H1	0.7635	−0.3891	1.0426	0.031*	
C2	0.7185 (4)	−0.5911 (3)	1.0460 (3)	0.0224 (6)	
H2	0.6933	−0.6409	1.1399	0.027*	
C3	0.7131 (4)	−0.6683 (3)	0.9661 (3)	0.0187 (5)	
C4	0.7549 (4)	−0.5898 (3)	0.8292 (3)	0.0242 (6)	
H4	0.7544	−0.6384	0.7709	0.029*	
C5	0.7970 (4)	−0.4404 (3)	0.7793 (3)	0.0275 (6)	
H5	0.8261	−0.3883	0.6856	0.033*	
C6	0.6512 (4)	−0.8286 (3)	1.0280 (3)	0.0204 (6)	
H6A	0.5805	−0.8497	1.1210	0.024*	
H6B	0.5619	−0.8407	0.9798	0.024*	
C7	0.9515 (4)	−0.9366 (3)	1.1004 (3)	0.0192 (5)	
H7A	0.8864	−0.9431	1.1912	0.023*	
H7B	1.0192	−0.8407	1.0532	0.023*	
C8	0.9075 (4)	−0.9347 (3)	0.8919 (3)	0.0196 (5)	
H8A	0.9749	−0.8389	0.8415	0.024*	
H8B	0.8124	−0.9387	0.8443	0.024*	
C11	0.8099 (3)	0.2813 (3)	0.5227 (2)	0.0170 (5)	
C12	0.6437 (3)	0.3901 (3)	0.5118 (2)	0.0161 (5)	
C13	0.4741 (4)	0.3639 (3)	0.6092 (2)	0.0172 (5)	
C14	0.6677 (4)	0.5255 (3)	0.4034 (3)	0.0178 (5)	
H14	0.7827	0.5429	0.3367	0.021*	
C15	0.4396 (3)	0.2173 (3)	0.7278 (3)	0.0176 (5)	

### Atomic displacement parameters (Å^2^)

**Table:**

	*U*^11^	*U*^22^	*U*^33^	*U*^12^	*U*^13^	*U*^23^
Co1	0.0153 (3)	0.0153 (3)	0.0161 (3)	0.00250 (18)	−0.0074 (2)	−0.0044 (2)
O1	0.0192 (9)	0.0174 (9)	0.0221 (9)	0.0051 (7)	−0.0094 (8)	−0.0073 (8)
O1W	0.0222 (10)	0.0262 (11)	0.0285 (10)	0.0026 (8)	−0.0124 (9)	−0.0074 (9)
O2	0.0238 (10)	0.0250 (10)	0.0270 (10)	0.0084 (8)	−0.0149 (8)	−0.0123 (8)
O3	0.0281 (10)	0.0165 (9)	0.0198 (10)	−0.0002 (8)	−0.0011 (8)	−0.0031 (8)
O4	0.0277 (11)	0.0174 (10)	0.0351 (12)	0.0074 (8)	0.0045 (9)	0.0008 (9)
O5	0.0186 (9)	0.0162 (9)	0.0184 (9)	0.0007 (7)	−0.0045 (8)	−0.0049 (7)
O6	0.0165 (9)	0.0201 (10)	0.0232 (10)	0.0033 (7)	−0.0082 (8)	−0.0090 (8)
N1	0.0265 (13)	0.0193 (12)	0.0329 (14)	0.0013 (9)	−0.0061 (11)	−0.0042 (10)
N2	0.0187 (11)	0.0141 (10)	0.0169 (10)	0.0003 (8)	−0.0062 (9)	−0.0033 (8)
C1	0.0251 (14)	0.0242 (14)	0.0303 (15)	0.0026 (11)	−0.0081 (12)	−0.0125 (12)
C2	0.0236 (14)	0.0207 (14)	0.0211 (13)	0.0019 (11)	−0.0074 (11)	−0.0052 (11)
C3	0.0156 (12)	0.0184 (13)	0.0207 (13)	0.0023 (9)	−0.0067 (10)	−0.0051 (10)
C4	0.0260 (14)	0.0226 (14)	0.0211 (13)	0.0030 (11)	−0.0072 (11)	−0.0049 (11)
C5	0.0297 (15)	0.0211 (14)	0.0216 (14)	0.0035 (11)	−0.0048 (12)	0.0004 (11)
C6	0.0181 (13)	0.0189 (13)	0.0212 (13)	0.0013 (10)	−0.0060 (11)	−0.0040 (10)
C7	0.0216 (13)	0.0195 (13)	0.0175 (12)	0.0017 (10)	−0.0089 (10)	−0.0060 (10)
C8	0.0204 (13)	0.0207 (13)	0.0176 (12)	0.0027 (10)	−0.0080 (10)	−0.0058 (10)
C11	0.0149 (12)	0.0135 (12)	0.0155 (12)	0.0013 (9)	−0.0016 (10)	0.0002 (9)
C12	0.0167 (12)	0.0132 (12)	0.0172 (12)	0.0022 (9)	−0.0070 (10)	−0.0034 (10)
C13	0.0178 (12)	0.0137 (12)	0.0177 (12)	0.0020 (9)	−0.0063 (10)	−0.0026 (10)
C14	0.0163 (12)	0.0160 (12)	0.0179 (12)	0.0013 (9)	−0.0052 (10)	−0.0027 (10)
C15	0.0142 (12)	0.0165 (12)	0.0196 (13)	0.0008 (9)	−0.0063 (10)	−0.0032 (10)

### Geometric parameters (Å, °)

**Table:**

Co1—O5	2.0631 (18)	C2—H2	0.9500
Co1—O1	2.1150 (17)	C3—C4	1.392 (4)
Co1—O6	2.1184 (18)	C3—C6	1.507 (4)
O1—C11	1.274 (3)	C4—C5	1.379 (4)
O1W—H1WA	0.881 (18)	C4—H4	0.9500
O1W—H1WB	0.885 (17)	C5—H5	0.9500
O2—C11	1.246 (3)	C6—H6A	0.9900
O3—C15	1.266 (3)	C6—H6B	0.9900
O4—C15	1.244 (3)	C7—C8^i^	1.517 (4)
O5—H5A	0.875 (17)	C7—H7A	0.9900
O5—H5B	0.852 (17)	C7—H7B	0.9900
O6—H6C	0.857 (17)	C8—C7^i^	1.517 (4)
O6—H6D	0.863 (17)	C8—H8A	0.9900
N1—C5	1.334 (4)	C8—H8B	0.9900
N1—C1	1.340 (4)	C11—C12	1.510 (3)
N2—C7	1.490 (3)	C12—C14	1.391 (3)
N2—C8	1.495 (3)	C12—C13	1.397 (3)
N2—C6	1.502 (3)	C13—C14^ii^	1.395 (3)
N2—H2N	0.906 (18)	C13—C15	1.514 (3)
C1—C2	1.384 (4)	C14—C13^ii^	1.394 (3)
C1—H1	0.9500	C14—H14	0.9500
C2—C3	1.393 (4)		
			
O5—Co1—O5^iii^	180.0	C5—C4—C3	119.0 (3)
O5—Co1—O1	88.13 (7)	C5—C4—H4	120.5
O5^iii^—Co1—O1	91.87 (7)	C3—C4—H4	120.5
O5—Co1—O1^iii^	91.87 (7)	N1—C5—C4	123.7 (3)
O5^iii^—Co1—O1^iii^	88.13 (7)	N1—C5—H5	118.2
O1—Co1—O1^iii^	180.000 (1)	C4—C5—H5	118.2
O5—Co1—O6	91.72 (7)	N2—C6—C3	115.3 (2)
O5^iii^—Co1—O6	88.28 (7)	N2—C6—H6A	108.4
O1—Co1—O6	88.61 (7)	C3—C6—H6A	108.4
O1^iii^—Co1—O6	91.39 (7)	N2—C6—H6B	108.4
O5—Co1—O6^iii^	88.28 (7)	C3—C6—H6B	108.4
O5^iii^—Co1—O6^iii^	91.72 (7)	H6A—C6—H6B	107.5
O1—Co1—O6^iii^	91.39 (7)	N2—C7—C8^i^	109.5 (2)
O1^iii^—Co1—O6^iii^	88.61 (7)	N2—C7—H7A	109.8
O6—Co1—O6^iii^	180.000 (1)	C8^i^—C7—H7A	109.8
C11—O1—Co1	122.61 (16)	N2—C7—H7B	109.8
H1WA—O1W—H1WB	104 (3)	C8^i^—C7—H7B	109.8
Co1—O5—H5A	123.9 (19)	H7A—C7—H7B	108.2
Co1—O5—H5B	123 (2)	N2—C8—C7^i^	110.4 (2)
H5A—O5—H5B	105 (2)	N2—C8—H8A	109.6
Co1—O6—H6C	116 (2)	C7^i^—C8—H8A	109.6
Co1—O6—H6D	102 (2)	N2—C8—H8B	109.6
H6C—O6—H6D	112 (3)	C7^i^—C8—H8B	109.6
C5—N1—C1	117.3 (2)	H8A—C8—H8B	108.1
C7—N2—C8	109.8 (2)	O2—C11—O1	125.8 (2)
C7—N2—C6	113.4 (2)	O2—C11—C12	117.0 (2)
C8—N2—C6	112.9 (2)	O1—C11—C12	117.1 (2)
C7—N2—H2N	102 (2)	C14—C12—C13	119.4 (2)
C8—N2—H2N	111.8 (19)	C14—C12—C11	117.6 (2)
C6—N2—H2N	106.2 (19)	C13—C12—C11	122.9 (2)
N1—C1—C2	123.1 (3)	C14^ii^—C13—C12	119.4 (2)
N1—C1—H1	118.5	C14^ii^—C13—C15	119.1 (2)
C2—C1—H1	118.5	C12—C13—C15	121.4 (2)
C1—C2—C3	119.2 (3)	C12—C14—C13^ii^	121.1 (2)
C1—C2—H2	120.4	C12—C14—H14	119.4
C3—C2—H2	120.4	C13^ii^—C14—H14	119.4
C4—C3—C2	117.7 (2)	O4—C15—O3	123.5 (2)
C4—C3—C6	121.9 (2)	O4—C15—C13	118.8 (2)
C2—C3—C6	120.3 (2)	O3—C15—C13	117.7 (2)
			
O5—Co1—O1—C11	54.13 (19)	C7—N2—C8—C7^i^	59.2 (3)
O5^iii^—Co1—O1—C11	−125.86 (19)	C6—N2—C8—C7^i^	−173.3 (2)
O6—Co1—O1—C11	145.90 (19)	Co1—O1—C11—O2	21.7 (3)
O6^iii^—Co1—O1—C11	−34.10 (19)	Co1—O1—C11—C12	−161.93 (16)
C5—N1—C1—C2	−0.8 (4)	O2—C11—C12—C14	93.1 (3)
N1—C1—C2—C3	−0.4 (4)	O1—C11—C12—C14	−83.6 (3)
C1—C2—C3—C4	1.2 (4)	O2—C11—C12—C13	−82.8 (3)
C1—C2—C3—C6	−174.9 (2)	O1—C11—C12—C13	100.5 (3)
C2—C3—C4—C5	−0.8 (4)	C14—C12—C13—C14^ii^	−0.5 (4)
C6—C3—C4—C5	175.2 (2)	C11—C12—C13—C14^ii^	175.3 (2)
C1—N1—C5—C4	1.2 (4)	C14—C12—C13—C15	178.9 (2)
C3—C4—C5—N1	−0.4 (4)	C11—C12—C13—C15	−5.3 (4)
C7—N2—C6—C3	59.1 (3)	C13—C12—C14—C13^ii^	0.5 (4)
C8—N2—C6—C3	−66.5 (3)	C11—C12—C14—C13^ii^	−175.6 (2)
C4—C3—C6—N2	81.0 (3)	C14^ii^—C13—C15—O4	156.2 (3)
C2—C3—C6—N2	−103.2 (3)	C12—C13—C15—O4	−23.2 (4)
C8—N2—C7—C8^i^	−58.7 (3)	C14^ii^—C13—C15—O3	−21.5 (4)
C6—N2—C7—C8^i^	174.0 (2)	C12—C13—C15—O3	159.2 (2)

Symmetry codes: (i) −*x*+2, −*y*−2, −*z*+2; (ii) −*x*+1, −*y*+1, −*z*+1; (iii) −*x*+2, −*y*, −*z*+1.

### Hydrogen-bond geometry (Å, °)

**Table:**

*D*—H···*A*	*D*—H	H···*A*	*D*···*A*	*D*—H···*A*
O1W—H1WA···O6^iv^	0.88 (2)	2.38 (3)	2.997 (3)	128 (3)
O1W—H1WB···O1^iv^	0.89 (2)	1.88 (2)	2.764 (3)	174 (3)
O5—H5A···N1	0.88 (2)	1.87 (2)	2.739 (3)	177 (3)
O5—H5B···O4	0.85 (2)	1.87 (2)	2.697 (3)	163 (3)
O6—H6C···O1W	0.86 (2)	1.92 (2)	2.753 (3)	165 (3)
O6—H6D···O2^iii^	0.86 (2)	1.81 (2)	2.624 (3)	158 (3)
N2—H2N···O3^v^	0.91 (2)	1.73 (2)	2.630 (3)	171 (3)

Symmetry codes: (iv) −*x*+1, −*y*, −*z*+1; (iii) −*x*+2, −*y*, −*z*+1; (v) −*x*+1, −*y*−1, −*z*+2.
